# AI competence and sentiment: a mixed-methods study of attitudes and open-ended reflections

**DOI:** 10.3389/frai.2025.1658791

**Published:** 2025-09-24

**Authors:** Gatis Lāma, Agnese Lastovska

**Affiliations:** ^1^Faculty of Social Sciences, Riga Stradins University, Riga, Latvia; ^2^University of Latvia, Riga, Latvia

**Keywords:** artificial intelligence, AI competence, sentiment analysis, thematic analysis, AI literacy

## Abstract

As artificial intelligence (AI) technologies become increasingly integrated into everyday life, understanding how the public perceives and interacts with AI is essential for fostering responsible and secure adoption. This study investigates the relationship between self-assessed AI competence, trust in AI-generated content, and sentiment toward AI among public and private sector employees in Latvia. Using a mixed-methods approach, the research combines quantitative survey data with open-ended qualitative responses to explore how demographic factors influence AI-related perceptions. Results reveal that although participants rate their AI competence and trust relatively highly, a significant portion of respondents either do not use AI or use it only for simple tasks. Sentiment toward AI is generally positive but often neutral, indicating that public attitudes are still forming. Statistically significant differences in AI competence were found across gender, age, and work sector, while trust in AI varied by education and age. Sentiment remained consistent across groups. Importantly, AI competence was positively correlated with trust, which in turn correlated with sentiment. Thematic analysis identified concerns about risk assessment, ethical implications, and the uncertain role of AI in daily life. The study underscores the need to enhance AI literacy and critical evaluation skills to ensure informed trust and societal resilience. These findings inform future strategies for public education, workforce training, and digital security policy in the context of accelerating AI adoption.

## Introduction

While the term “artificial intelligence” continues to evolve, major institutions offer working definitions that help frame this study. The OECD defines AI as machine-based systems that make predictions or decisions to affect real or virtual environments (Berryhill et al., 2019). The European Commission views AI as autonomous systems that pursue goals based on environmental input (European Commission, 2019). These evolving definitions underscore AI’s complexity and its wide-ranging implications for different sectors of society (Lāma and Lastovska, 2025).

Artificial intelligence (AI) has rapidly become embedded in everyday life, powering technologies from virtual assistants to medical diagnostics (Faverio and Tyson, 2023). As AI systems increasingly assist with decisions and automate services, the public’s trust in these systems has emerged as an important factor in their widespread adoption (Afroogh et al., 2024). Trust (or distrust) acts as a “regulator” of AI’s diffusion: people are more likely to embrace AI applications they trust, and conversely, distrust can significantly slow down adoption (Afroogh et al., 2024). Indeed, realizing AI’s benefits for society requires maintaining public confidence that AI is developed and used responsibly. Sustained acceptance and effective use of AI in society are founded on this trust (Gillespie et al., 2023). In other words, if users do not trust AI technologies—whether due to concerns about bias, privacy, or reliability—they may reject even beneficial AI solutions, undermining the positive impact these systems could have.

Importantly, trust and competence in AI are not only matters of ethical adoption or innovation—they are increasingly viewed as key pillars of digital and national security. AI technologies now underpin essential sectors such as cybersecurity, infrastructure monitoring, healthcare, finance, and law enforcement. Owing to the rapid advances in information and communication technologies (ICT) and their increasing pervasiveness, disingenuous information can now be produced easily and in a realistic format, and its dissemination to a targeted audience occurs at an unparalleled speed and scale—including through AI techniques (Bontridder and Poullet, 2021). This significantly amplifies the threat of misinformation, propaganda, and influence operations in the digital public sphere.

Moreover, AI-driven systems themselves are increasingly susceptible to cybercriminal activities, including data breaches, adversarial assaults, and zero-day vulnerabilities (Almaiah, 2025). Public misunderstanding or misjudgment of these technologies can create vulnerabilities—ranging from inadequate privacy practices to exposure to manipulation and cyber threats. A population lacking AI competence may fall victim to AI-driven fraud, exploitation, or disinformation campaigns, making societal resilience against digital threats harder to maintain. Thus, advancing public competence in AI is not just a matter of inclusion or fairness—it is a strategic necessity in a world where both everyday decisions and national defense increasingly depend on automated systems.

Public trust in AI does not develop in a vacuum; it is closely intertwined with people’s understanding of and confidence in AI. Self-assessed AI competence (often termed AI literacy or self-efficacy with AI) refers to how knowledgeable and capable individuals feel about AI. Research suggests that this competence plays a significant role in shaping trust and attitudes. For example, a recent study found that individuals with higher AI literacy tend to exhibit greater trust in AI technologies across various practical scenarios (Huang and Ball, 2024). Those with advanced understanding of AI were consistently more trusting of AI systems, whereas people with only moderate familiarity showed increased skepticism, especially in high-stakes contexts like healthcare or transportation (Huang and Ball, 2024). Similarly, a large cross-cultural survey reported that higher self-efficacy and competency in AI correlate with more positive attitudes toward AI (Naiseh et al., 2025). In that study, feeling more knowledgeable and capable with AI went hand-in-hand with greater confidence and optimism about using AI (Naiseh et al., 2025). These findings support the notion that when people understand how AI works, its limitations, and its potential, they are more likely to trust the technology and respond to it positively, rather than with fear or confusion.

Broader public sentiment towards AI encompasses a spectrum from enthusiasm and curiosity to wariness and fear. Recent surveys indicate a cautious optimism among many communities. Globally, a slight majority now believes AI’s benefits will outweigh its drawbacks (Maslej et al., 2024), yet concerns remain high about specific risks and ethical issues. In the United States, for instance, 52% of adults report feeling more concerned than excited about the growing role of AI in daily life (only 10% are more excited than concerned) (Faverio and Tyson, 2023). Such cautious sentiment underscores why public competence and trust are so important for the ethical and effective deployment of AI. If people lack understanding of AI, they may overestimate its threats or underestimate legitimate risks, leading to either undue fear or unwarranted overtrust. Both scenarios carry ethical implications—but also security consequences: fear can hinder the adoption of AI tools essential for public safety, while blind overtrust can expose individuals to harm from unverified, malicious, or biased AI systems. Improving the public’s AI competence is therefore seen as a critical step toward addressing these issues. An AI-literate public is better equipped to interpret AI outputs and intentions, which fosters informed trust, more measured expectations, and safer user behavior (Kumar et al., 2025). In fact, studies note that enhancing AI literacy among users encourages trust in AI-driven tools and improves acceptance of innovative applications (e.g., AI in telemedicine or autonomous vehicles) (Kumar et al., 2025). By cultivating understanding and addressing people’s concerns, stakeholders can ensure AI is deployed in a manner that aligns with societal values, adheres to ethical standards, and reinforces digital resilience and security.

While there is growing recognition of the links between AI literacy, trust, and attitudes, these factors have typically been studied in isolation. On one hand, numerous studies have measured public attitudes toward AI using structured questionnaires and scales—for example, instruments like the Attitude Towards AI Scale or the General Attitudes towards AI Scale capture people’s general positivity or negativity toward AI (Şahin and Yıldırım, 2024). On the other hand, separate research streams have examined AI literacy or competence, including interventions to improve people’s understanding of AI, and assessed outcomes such as user behavior or basic trust levels (Gillespie et al., 2025). Most prior surveys rely on predefined statements, which may not fully reflect the nuances of how people feel about AI. Open-ended sentiment—the emotions and opinions people freely express about AI—is an under-explored dimension in quantifying public attitudes. Understanding this richer sentiment alongside quantitative measures of trust and competence is important because it can reveal why people hold certain attitudes. For instance, two individuals might both report low trust in AI on a survey, but an open-ended response could show that one fears AI stealing jobs while the other is concerned about privacy violations. Without analyzing such qualitative sentiments, researchers and policymakers might miss context critical for addressing public concerns. Therefore, a gap exists in the literature: integrative studies that examine how people’s AI competence (literacy) relates to their trust in AI, and how both relate to the sentiments (positive or negative feelings) people articulate about AI. Addressing this gap is important for developing a holistic understanding of public interaction with AI. By linking competence, trust, and sentiment, the present research aims to shed light on how educating the public about AI might influence their trust and emotional responses, and vice versa, ultimately informing strategies for more responsible AI design, user education, and policy-making that proactively safeguards public confidence and digital security.

Significant development processes nowadays take place in workplaces. Despite the longstanding presence of AI in academic and technological discourse, its adoption in the public sector has consistently lagged behind that of the private sector (Wirtz et al., 2018). While private enterprises have proactively responded through investment in AI-focused training, organizational learning, and cultural transformation (Lāma and Lastovska, 2025), public sector institutions are only beginning to adapt to the new digital paradigm. They must now confront not only technical challenges but also organizational, ethical, and attitudinal barriers that shape AI integration (Lāma and Lastovska, 2025). This study focuses on Latvia as a representative case of a small European country navigating these complex demands. Latvia’s national strategy emphasizes innovation and e-governance, yet its public sector continues to face significant limitations in workforce capacity, infrastructure, and AI readiness (Lāma and Lastovska, 2025). These dynamics make Latvia a compelling site for examining both the drivers and obstacles to effective AI adoption, especially among public employees.

The aim of the research is to examine how individuals’ self-assessed AI competence relates to their trust in AI-generated content and their emotional sentiment toward AI, with the goal of identifying correlations, differences across socio-demographic groups, and thematic patterns in qualitative reflections.

Research questions

1. Does the Latvian population possess adequate AI competence, trust in AI-generated content, and positive sentiment toward AI?
2. Are AI competence, trust in AI-generated information, and sentiment toward AI interconnected?
3. How does AI Competence, trust in information generated by AI and sentiment differ across various socio-demographic groups?
4. What themes emerge in people’s opinions about AI?

### Theoretical framework

Researchers have increasingly focused on AI literacy and competence frameworks to define what it means to be “AI-competent.” Many frameworks adapt classic educational taxonomies (e.g., Bloom’s taxonomy) to the AI context (Carolus et al., 2023; Lāma and Lastovska, 2025), mirroring the progression from basic knowledge to higher-order skills such as creation and evaluation, and explicitly incorporating an ethics dimension—underscoring that ethical awareness is a crucial component of AI competence (Almatrafi et al., 2024).

At the core of this study lies a conceptual triad: AI competence, trust in AI, and sentiment toward AI. In this model, competence—defined as individuals’ self-assessed ability to understand, use, and critically evaluate AI systems—forms the foundation for trust, which in turn shapes overall sentiment. This theoretical framing draws on Mayer et al.’s (1995) trust model, which identifies competence as a key antecedent of trust, and is reinforced by empirical studies in human–AI interaction (Aaker et al., 2010; Glikson and Woolley, 2020; Dang and Li, 2025).

From a psychological perspective, familiarity with a technology often breeds comfort and trust—up to a point. A consistent theme in recent literature is that higher AI literacy often correlates with greater confidence, perceived usefulness, and positive attitudes toward AI (Bewersdorff et al., 2025), yet this relationship is not uniformly linear. Some highly knowledgeable users develop what Pan et al. (2025) term “informed skepticism,” setting a higher bar for trust when they are aware of issues such as bias, lack of transparency, or error-prone performance. This dual pattern aligns with technology acceptance models, where competence can enhance perceived usefulness and reduce anxiety (Nillos, 2016; Abbad, 2021), but also with the notion of “cautious critics” who combine strong knowledge with vigilant oversight (Bewersdorff et al., 2025).

Trust functions as a mediating mechanism in this framework. Users cognitively evaluate AI capabilities—assessing reliability, accuracy, and functionality (Hertel et al., 2006)—and these evaluations directly shape trust. High performance and transparency tend to enhance trust, while unpredictability or errors diminish it (Chandra et al., 2022; Hancock et al., 2011). Expectancy-disconfirmation theory further explains how trust influences sentiment: when AI meets or exceeds expectations for accuracy and fairness, trust reinforces positive sentiment; when it fails, trust erodes and sentiment turns negative (Bhattacherjee, 2001; Oliver, 1980; Balakrishnan and Dwivedi, 2021).

Sentiment, in this context, captures the emotional and attitudinal orientation toward AI—ranging from enthusiasm to apprehension—and is shaped by both cognitive trust judgments and personal values. Positive sentiment often follows favorable trust assessments, leading to acceptance, advocacy, and engagement (Afroogh et al., 2024). Conversely, low trust prompts skepticism, resistance, and heightened focus on perceived risks such as job loss, privacy violations, or unethical use (Acemoglu, 2021; Frey and Osborne, 2017).

**Figure 1:**
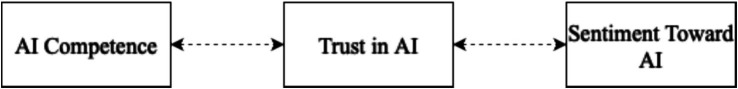
The conceptual model illustrating the proposed theoretical framework, where AI competence influences trust in AI, which in turn shapes sentiment toward AI.

Building on this literature, we propose a competence → trust → sentiment pathway as a robust, empirically grounded framework for understanding AI perception in workplace contexts (Figure 1).

Quantitatively, we assess whether the Latvian population possesses adequate AI competence, trust in AI-generated content, and positive sentiment toward AI, and whether these factors are interconnected. We also examine how competence, trust, and sentiment vary across socio-demographic groups, acknowledging that domain, perceived stakes, and individual values may influence attitudes. Qualitatively, we identify the themes that emerge in public and private sector employees’ reflections, illustrating how competence development can shift both trust levels and emotional orientations toward AI—from enthusiasm to caution—depending on knowledge depth, perceived risk, and application context.

### Sentiment and thematic analysis of open-ended AI perspectives

Beyond quantitative surveys, researchers have employed open-ended questions and text analysis to capture the nuances of how people talk and feel about AI. The advent of powerful Python-based natural language processing (NLP) tools has enabled large-scale analysis of free-text responses, social media posts, and interview transcripts. Typical methodologies include sentiment analysis – detecting whether text expresses a positive, negative, or neutral sentiment – and thematic analysis or topic modeling – discovering recurring themes and concerns in the content. These approaches provide a richer understanding of public discourse around AI, revealing not just what people know, but how they *feel* and *what issues* they frequently mention.

Automated sentiment analysis is often performed using Python libraries such as NLTK/VADER or transformer-based models. For example, Arboleda et al. (2024) analyzed ~39,000 tweets about ChatGPT using a combination of VADER (a lexicon method) and the NRC emotion lexicon, integrated with Python code, to quantify sentiment polarity and emotional tone. Their results showed a predominantly positive or neutral sentiment in the public’s early reactions to ChatGPT. Over half (54.4%) of tweets carried a positive tone, while only 17% were negative, and the rest neutral (Arboleda et al., 2024). Emotional analysis indicated that *trust, anticipation,* and *joy* were the most frequently expressed emotions on social media regarding ChatGPT (Arboleda et al., 2024) – suggesting an overall optimistic and hopeful public outlook at that time. Negative emotions like fear or anger were present but less common, reflecting that although some users voiced concerns or skepticism, the general vibe leaned optimistic. Another study of Twitter discourse by Koonchanok et al. (2024) likewise found neutral-to-positive overall sentiment, with negative sentiment actually decreasing over time as people became more familiar with ChatGPT. This temporal trend implies that initial worries may have been somewhat allayed as users saw more practical examples of AI’s capabilities (or simply grew accustomed to the technology).

To complement sentiment scores, researchers apply topic modeling and thematic coding to open-ended data, revealing what specific themes or issues dominate AI discussions. Using techniques like Latent Dirichlet Allocation (LDA) or BERTopic (in Python), studies have uncovered the main topics people associate with AI. In Arboleda et al.’s analysis of tweets, the key themes revolved around ChatGPT’s potential and utility – topics such as its use in education, its functionality in content creation, and its integration into search or marketing were prominent (Arboleda et al., 2024). Koonchanok et al. identified popular topics by month, with education, search engines, marketing, cybersecurity, and AI research (OpenAI itself) among the most discussed aspects of ChatGPT (Koonchanok et al., 2024). Notably, users tended to discuss AI in ways relevant to their own fields – for example, tech professionals talked about cybersecurity and coding applications, while teachers and academics discussed educational uses (Koonchanok et al., 2024). This indicates that people contextualize AI’s usefulness (or threats) within their domain of interest, which is an important consideration for thematic analysis of open-ended responses in surveys. If a study asks the public “What do you think about AI?,” a student might mention AI helping with homework, whereas a content creator might mention AI in art or writing – each highlighting different hopes or concerns.

Across various sentiment analyses of AI discourse, several recurring themes emerge. Ethical concerns are one major theme: people frequently raise issues of *bias, fairness, privacy,* and the need for responsible AI use (Vesely and Kim, 2024). For instance, in a qualitative interview study on AI in mobile health apps, end-users consistently brought up trust and ethics – they wanted to know that AI decisions were endorsed by professionals, that their personal data was safe, and that the AI’s recommendations were explainable (Ryan et al., 2025). Misinformation is another prevalent worry: with the rise of deepfakes and AI-generated content, many respondents fear AI could *“supercharge”* the spread of false information (Yan et al., 2025). In fact, a 2024 survey in Europe and the US found that concerns about AI-driven misinformation and manipulation were among the top reasons the public supports stricter AI oversight (Vesely and Kim, 2024). This indicates that even when people express positive sentiment about AI’s capabilities, there is an undercurrent of caution about AI being misused to deceive or misinform.

On the positive side, usefulness and productivity form a key theme in AI discourse. Open-ended feedback often highlights AI’s *efficiency and problem-solving potential*. Many respondents describe AI as a powerful tool – for example, mentioning how generative AI can save time in drafting documents, or how AI analytics can improve decision-making in business. Such comments reflect an appreciation of AI’s practical benefits, aligning with the high proportion of joyful or anticipatory sentiments on social media (Arboleda et al., 2024). Especially among those who have used AI tools, sentiments of *amazement* at what AI can do are common, as are stories of AI yielding valuable insights or creative outputs. These positive narratives feed into a broader social sentiment that AI, if harnessed well, could augment human capabilities in many domains.

Finally, we see references to emotional and social impacts of AI in the qualitative data. Some people express *anxiety or concern* about how AI might affect human relationships, jobs, or society at large. For instance, open-ended survey responses and interviews have noted fears of AI causing unemployment (a social impact) or reducing human contact (e.g., “Will AI replace my teacher or my doctor?” indicating an emotional concern about losing human touch) (Vesely and Kim, 2024). Others, however, voice *excitement* that AI could handle mundane tasks and free up humans for more creative or interpersonal work – an optimistic social vision. Emotions like anticipation and curiosity suggest that many are eagerly watching how AI evolves and what it means for the future of work, education, and daily life (Arboleda et al., 2024). In the public Twitter discourse, *trust* emerged as a frequently expressed emotion regarding ChatGPT (Arboleda et al., 2024), implying a notable portion of users felt comfortable relying on it – an interesting social indicator of AI’s integration into everyday life. Yet, trust in this context may be tentative and contingent on AI *meeting certain expectations* (e.g., being accurate, unbiased). As one qualitative study concluded, users often draw a line between where AI is valuable and where it is not: for example, participants were willing to extend a degree of trust or “empathy” to AI in health apps if the AI proved helpful, but they remained wary of AI that lacked explainability or accountability (Ryan et al., 2025).

Examples of studies using sentiment analysis and thematic analysis on open-ended AI discussions. These studies employed Python-based NLP tools to quantify sentiment (positive/negative emotion) and extract prevalent themes. Common findings include a generally positive public sentiment toward new AI tools like ChatGPT (Arboleda et al., 2024), coupled with prominent discussion of ethical concerns, trust, and AI’s practical uses across different communities (Ryan et al., 2025).

The literature reveals a multifaceted relationship between individuals’ self-assessed AI competence and their attitudes toward AI, as well as rich insights from sentiment analysis of how people express their hopes and concerns about AI in their own words. On the one hand, higher AI literacy – whether measured through structured frameworks (Bloom-inspired cognitive skills, ethical awareness, etc.) or simple self-report familiarity – often correlates with more positive attitudes, greater trust in AI systems, and higher self-efficacy in using AI (Bewersdorff et al., 2025). People who understand AI’s capabilities and limits tend to appreciate its usefulness and are willing to integrate it into their work or studies. On the other hand, research also cautions that knowledge brings nuance: competent users might approach AI with informed skepticism, identifying pitfalls that less savvy users overlook (Pan et al., 2025). Thus, while lack of knowledge can breed unfounded fears or unrealistic expectations, extensive knowledge can breed a healthy caution that tempers over-optimism. Effective AI education should strive to produce users who are both confident and critical – trusting AI where warranted but mindful of ethical and reliability issues.

Analyses of open-ended responses reinforce that attitudes toward AI are not monolithic. Through sentiment and thematic analysis, we see a public discourse that is broadly positive about AI’s potential yet continually interrogating its implications. Key themes like ethics, trust, misinformation, and social impact recur across studies, indicating these are universal touchstones in the AI debate. Even as many marvel at AI’s innovative applications, they simultaneously worry about privacy, bias, and the loss of human touch or jobs (Ryan et al., 2025). Sentiment analysis with Python tools has shown that excitement (joy, anticipation) and optimism (trust) currently outweigh fear in many forums (Arboleda et al., 2024), but the margin of public trust is conditional. Transparency, education, and responsible AI practices will be crucial in maintaining and improving positive attitudes.

Overall, the literature since 2018 paints a picture of a society in the early stages of grappling with AI: people are learning about AI (building competence) and forming attitudes in real-time, while researchers develop better instruments to measure these constructs. There is a clear call for more studies that link objective AI literacy to subjective attitudes – and for interventions that boost both. By understanding the correlation (or lack thereof) between what people *think they know* about AI and how they *feel* about it, stakeholders can design educational programs that not only impart knowledge but also address misconceptions and fears. Moreover, incorporating sentiment and thematic analyses into AI perception research provides a holistic view: it captures not just survey tick-box responses, but the genuine voices of users – their excitement, reservations, suggestions, and lived experiences with AI. Such comprehensive insight is invaluable for informing AI design, policy, and education that resonate with public values. The evidence reviewed here underscores that improving AI competence (from basic literacy to advanced skills) is likely to foster more empowered and nuanced attitudes toward AI, which in turn can lead to more effective and ethical use of AI in society. As we move forward, continued monitoring of both the knowledge and the sentiments surrounding AI will be key to ensuring that the evolution of AI technology remains aligned with human needs, expectations, and well-being (Ryan et al., 2025).

### Security and AI

The competence of citizens in understanding and using artificial intelligence (AI) technologies has direct and far-reaching implications for individual, societal, and national security. As AI systems become increasingly integrated into daily life, the ability of citizens to engage with these technologies in a knowledgeable and responsible manner is significant for mitigating associated security risks.

One of the factors influencing the security impact of AI is public perception. Effective communication about AI’s benefits and risks is essential. Miscommunication or lack of transparency can exacerbate public fears and hinder the acceptance of AI technologies (Goicochea Parks et al., 2024; Brauner et al., 2023). Citizens’ emotional responses to AI—such as feelings of dread or perceived lack of control—shape how they assess and respond to AI-related risks. These perceptions are not formed in isolation but are influenced by broader cognitive and social factors such as trust in scientists, susceptibility to conspiracy theories, and beliefs about the impact of technological change on employment (Romualdi et al., 2025). These beliefs can hinder the public’s ability to critically assess the opportunities and threats posed by AI, potentially leading to either complacency or unwarranted fear—both of which are detrimental to security.

Trust in AI systems and the implementation of privacy-protective behaviors are significant predictors of AI adoption and safe usage. Notably, online skills alone do not significantly influence whether individuals use AI-based services, suggesting that trust and perceived control over personal data play a more crucial role (Jang, 2023). A lack of understanding of these dimensions may lead individuals to unknowingly compromise their privacy or avoid using beneficial AI services due to unfounded concerns, both of which can have security consequences.

The ethical implications of AI—such as algorithmic biases, opaque decision-making, and privacy concerns—further highlight the need for public competence. Ensuring fairness, accountability, and transparency in AI applications is essential for maintaining public trust and preventing social harm (Blancaflor et al., 2024; Suo et al., 2024). When citizens lack the knowledge to identify or question unethical AI behavior, the risk of abuse and unchecked harm increases, potentially eroding democratic norms and individual rights.

AI also plays a dual role in cybersecurity. On one hand, it enhances threat detection and response capabilities; on the other hand, it introduces new vectors of attack, such as AI-powered phishing or autonomous malware. This duality underscores the importance of citizen awareness and preparedness. Informed citizens are more likely to recognize, report, and protect against such threats, while uninformed individuals may become easy targets or even unwitting enablers of cyberattacks (Jadoun et al., 2025).

The human element remains a critical vulnerability in cybersecurity. Individual perceptions of vulnerability and control strongly influence behavior in digital environments. When citizens are unaware of the risks posed by AI technologies or lack the skills to manage them, they become more susceptible to manipulation, data breaches, and exploitation (Debb and McClellan, 2021; Abdel Rahman et al., 2024). Educating the public about these risks and how to respond effectively is thus a central component of national cybersecurity strategies.

Moreover, public education and awareness-raising campaigns can help dispel myths and correct misunderstandings that fuel resistance to AI. Addressing misinformation, conspiracy thinking, and skepticism toward scientific authority is vital to building a resilient and informed society (Romualdi et al., 2025). Such educational efforts must go beyond technical training to include ethical, social, and political dimensions of AI competence.

The lack of AI competence among citizens not only limits their ability to benefit from AI but also increases their exposure to security risks. These include vulnerabilities to cyber threats, misjudgment of AI systems’ capabilities and intentions, and poor data privacy practices. Enhancing citizens’ understanding of AI through targeted education, public engagement, and interdisciplinary training can empower them to make informed decisions, adopt protective behaviors, and actively contribute to a secure digital society.

Higher trust in government and scientists correlates with more favorable perceptions of AI, which can support the secure implementation of AI technologies in critical areas such as digital infrastructure, data protection, and public safety (Wang, 2025). Conversely, lower trust can heighten perceived risks, leading to resistance that undermines the adoption of AI systems designed to strengthen cybersecurity or enhance threat detection. When public skepticism grows, it can create vulnerabilities by weakening cooperation with AI-driven security measures and increasing exposure to disinformation and manipulation.

Media portrayal of AI plays a significant role in shaping these perceptions. Positive coverage can improve public trust and foster more responsible engagement with AI, whereas negative or sensationalist reporting may intensify fears, misconceptions, and mistrust (Moriniello et al., 2024). As studies show, the polarization of media sentiment—marked by rising extremes in both positive and negative views—can lead to fragmented public opinion, complicating security policy development and reducing societal resilience to AI-driven threats (Moriniello et al., 2024). Enhancing AI competence through education and public engagement is essential for mitigating these risks. A better-informed population is less likely to perceive AI as a mysterious or uncontrollable force and more likely to participate in secure, privacy-conscious behaviors. Promoting AI literacy enables individuals to critically assess information, recognize threats, and make informed decisions that support both personal and collective digital security (Brauner et al., 2023; Kendall Roundtree, 2024).

### Methodology

Research data were collected with online survey tool QuestionPro. The survey consisted of socio-demographic questions, AI competence statements, statements about attitude toward AI and ethics, educational opportunities in the workplace as well as comment field (Appendix A). This research deals only with AI competence, Trust in AI and comment section. AI competence was measured with Likerts scale adapted from Bloom’s taxonomy (0—I have not heard about the appropriate AI tools, 1—I have heard something about the appropriate AI tools but have not used them, 2—I have heard about the appropriate AI tools and know how to use them, 3—I have used the appropriate AI tools for simple tasks, 4—I deliberately analyze my daily work and select the most appropriate AI tools, 5—I evaluate and combine different AI tools, 6—I improve the appropriate AI tools or integrate them into other systems). Trust in information generated by AI was measured with a 4-point Likerts scale (4—Yes, 3—Rather yes, 2—Rather no, 1—No). This study draws on Bloom’s taxonomy – a widely used framework that classifies cognitive objectives from basic to advanced: remember, understand, apply, analyse, evaluate, and create. In our study, Bloom’s levels were adapted to measure the depth of engagement with AI tools through a seven-point scale to ensure that competence is measured not just as binary (yes/no) usage but as a gradient of mastery (Anderson et al., 2001). As the use of AI had become relatively recent in the wider community, consideration was given to embrace a broad view of AI competence through a single-item scale, which can improve the clarity of the issue and reduce respondents’ confusion (Allen et al., 2022). The comment section without any specific question was also included in the questionnaire for respondents who wished to share additional thoughts. Surprisingly, 486 comments were submitted. Given their richness and relevance, it was decided to analyze these responses.

The survey was distributed via email. Public sector employees’ email addresses were gathered from official municipalities websites, and an email with an invitation to participate, along with a link to the online survey, was sent to all employee emails. In total, 11,302 emails were sent and 1,557 public sector employees participated in the study. Additionally, an invitation to participate in the survey was published on researchers Facebook pages and in news portal jauns.lv which generated additional 156 participants (Table 1).

**Table 1:** Socio-demographic profile of study participants.

Age	Count
18–29	124
30–39	351
40–49	469
50–59	505
60–74	255
75+	3
Do not want to specify	6
Gender	
Male	298
Female	1,405
Do not want to specify or other	10
Education	
Basic education	3
Secondary education	58
Vocational education	98
Higher education	1,554
Work sector	
Private sector	154
Public sector	1,559
Type of populated point	
Capital (Rīga)	370
Cities (Ventspils, Liepāja, Jūrmala, Jelgava, Rēzekne, Daugavpils, Valmiera, Ogre, Jēkabpils)	355
Other towns	595
Countryside	393

In total, there are 1.85 million inhabitants in Latvia; therefore, with a 95% confidence level, the sample’s margin of error is 2.36. Additionally, power analysis was conducted and results indicated that sample size is sufficient. However, the data is skewed towards female respondents, with much higher activity coming from those in the public sector, where more women are employed compared to men; nevertheless, they men are still underrepresented.

Sentiment analysis for the comments section was carried out. First comments with incoherent messages or symbols were removed and then all comments that were intended as an answer to the caption of this section like “no,” “not,” “no comments” etc. were also removed. All the remaining 354 comments were translated into English with hugo.lv which is a language technology platform which provides automated translation services and is developed by the Latvian government, freely accessible to every resident of Latvia. Additionally, sentiment analysis scores were manually checked whether there are any scores that do not reflect the content of the comment.

Sentiment scores were calculated in Python with NLTK (Natural Language Toolkit) library VADER sentiment analysis tool (Hutto and Gilbert, 2014). For each comment sentiment score was calculated. Further, descriptive statistics were used. Spearman rank correlation test was carried out to find whether there is connection between AI competence, trust in AI-generated content and sentiment toward AI. Mann–Whitney *U* test and Kruskal-Wallis *H* test was carried out to determine whether there is a difference between AI competence, trust in AI-generated content and sentiment toward AI among different socio-demographic groups. To analyse the comment section, the qualitative data analysis software NVivo (release 15.2.1, 2019) was used. Two researchers conducted inductive coding to ensure research rigour. This process yielded four codes that showed the highest level of agreement between the researchers and had the most references. The comments were also examined using qualitative thematic analysis, which allowed for the identification of shared perspectives and emotional nuances that were not fully captured by the structured survey items.

The questionnaire was available for completion from July 6, 2024, to August 30, 2024, and the data were analyzed using SPSS version 29 and Microsoft Excel and Python version 3. The study adhered to all ethical research standards in accordance with the General Data Protection Regulation (GDPR). Participants completed the questionnaire anonymously, and participation was entirely voluntary. Approval for conducting this research was obtained from the Research Ethics Committee of Social Sciences and Humanities of the University of Latvia (Nr. 71-43/87).

## Results

AI competence was measured in a 7 point scale (from 0 to 6). Results indicate that participants perceive their AI competence as well developed (M = 3.46, SD = 1.09) as mean value is above scales average (Table 2).

**Table 2:** Self-assessments of participants AI competence, trust in AI and sentiment towards AI.

Item	Mean	Median	Std. dev	Skewness	Kurtosis
AI competence	3.46	4	1.09	0.09	−0.05
Trust in AI	2.38	2	0.63	−0.37	−0.54
Sentiment towards AI	0.20	0.18	0.35	−0.19	−0.22

**Figure 2:**
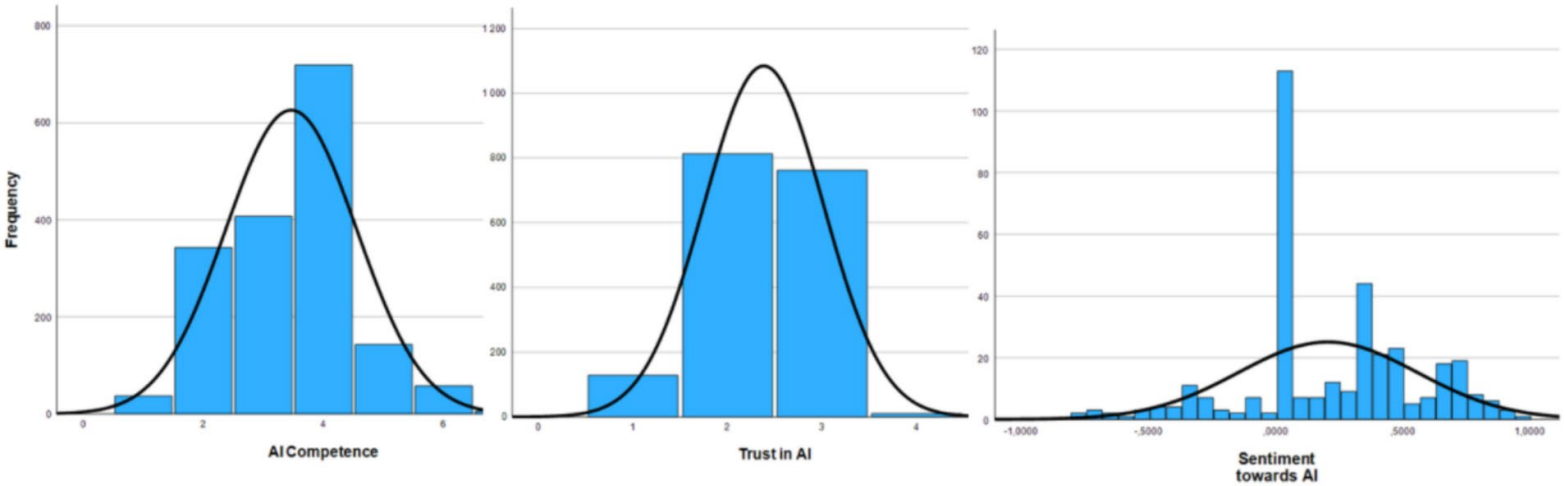
Participant AI competence, trust in AI and sentiment towards AI distribution.

Median (Mdn = 4) shows that half of the participants deliberately analyze their daily work and select the most appropriate AI tools. Trust in AI was measured in 4 point Likerts scale (from 1 to 4) and results indicate that more than half of respondents have indicated that they rather do not trust AI generated information. Participants’ scepticism of AI-generated information should not be necessarily seen as a bad outcome as AI can generate false information and from the security aspect complete belief in AI generated information would potentially pose further threats. Although the sentiment value is positive, it is relatively low, indicating that sentiment should rather be considered neutral. Analysis of assessment distribution indicates that most of the participants have measured their AI competences with 4 meaning that they deliberately analyze their daily work and select the most appropriate AI tools (Figure 2).

However, many respondents have measured their competence with 2 and 3 meaning that they have heard about appropriate AI tools or have tried to use them only for simple tasks. These respondents may be vulnerable to AI-generated misinformation, as they might lack the competence to recognize whether the information was created by AI and is therefore false. Based on the distribution of respondents’ answers about their trust in AI-generated information, it can be concluded that most tend to distrust AI. Nevertheless, a considerable portion of participants expressed moderate trust. Only a small group fully trusts or fully distrusts AI. Distribution of sentiment analysis indicates that most of the participants have neutral sentiment towards AI. That can be explained with the fact that for a lot of participants AI is relatively new and there is no sentiment towards AI formed yet. Data is skewed towards positive sentiment and it allows to conclude that participants have recognized the potential usefulness of AI.

Results of Spearman rank correlation test indicate that there is statistically significant moderate correlation between AI competence and trust in AI (r_s_ = 0.361) (Table 3).

**Table 3:** Spearman rank correlation between AI competence, trust in AI and sentiment towards AI.

Item	Correlation	AI competence	Trust in AI	Sentiment towards AI
AI competence	Correlation coefficient	1,000	0.361^**^	0.033
	*N*	1713	1713	354
Trust in AI	Correlation coefficient		1,000	0.164^**^
	*N*		1713	354
Sentiment towards AI	Correlation coefficient			1,000
	*N*			354

**Correlation is significant at the 0.01 level (2-tailed).

It indicates that greater familiarity with AI functions leads to higher trust in AI. This may suggest that increased experience with AI enhances participants’ ability to recognize potential flaws of AI and evaluate the credibility of results, thereby increasing their trust in the outcomes. Nevertheless, further research is necessary to explain the relationship between AI competence and attitudes toward it. There is also a statistically significant weak correlation between trust in AI and sentiment towards AI (r_s_ = 0.164). Both, trust and sentiment is part of the attitude. It indicates that sentiment which is more of a feeling and trust which indicates to be more of deliberate and rational attitude are connected. It is possible that positive sentiment towards AI can increase trust in information generated by AI and further increase willingness to increase one’s AI competence. However, there is no direct correlation between sentiment towards AI and AI competence. Further research is required to better understand the relationship between AI competence, trust in information generated by AI and sentiment towards AI.

To understand whether there are differences among different socio demographic groups Mann–Whitney U test or Kruskal-Wallis H test was conducted. Results indicate that there is statistically significant difference between male and female AI competence while trust in AI and sentiment towards AI by gender do not have statistically significant differences (Table 4).

**Table 4:** Comparison of participants AI competence, trust in AI and sentiment towards AI self-assessment rankings by gender (Mann–Whitney U test).

Item	Gender	Mean	Median	Std. dev	*N*	Mean Rank	Sum of Ranks	U	*P*	η^2^
AI competence	Male	3.67	4	1.07	298	946	281,764	181,477	<0.000	0.008
	Female	3.41	4	1.08	1,405	832	1,169,192			
Trust in AI	Male	2.39	2	0.67	298	861	256,687	206,554	0.686	<0.001
	Female	2.38	2	0.62	1,405	850	1,194,269			
Sentiment towards AI	Male	0.15	0	0.19	60	168	10,077	8,247	0.467	<0.001
	Female	0.21	0	0.18	292	178	52,051			

**Figure 3:**
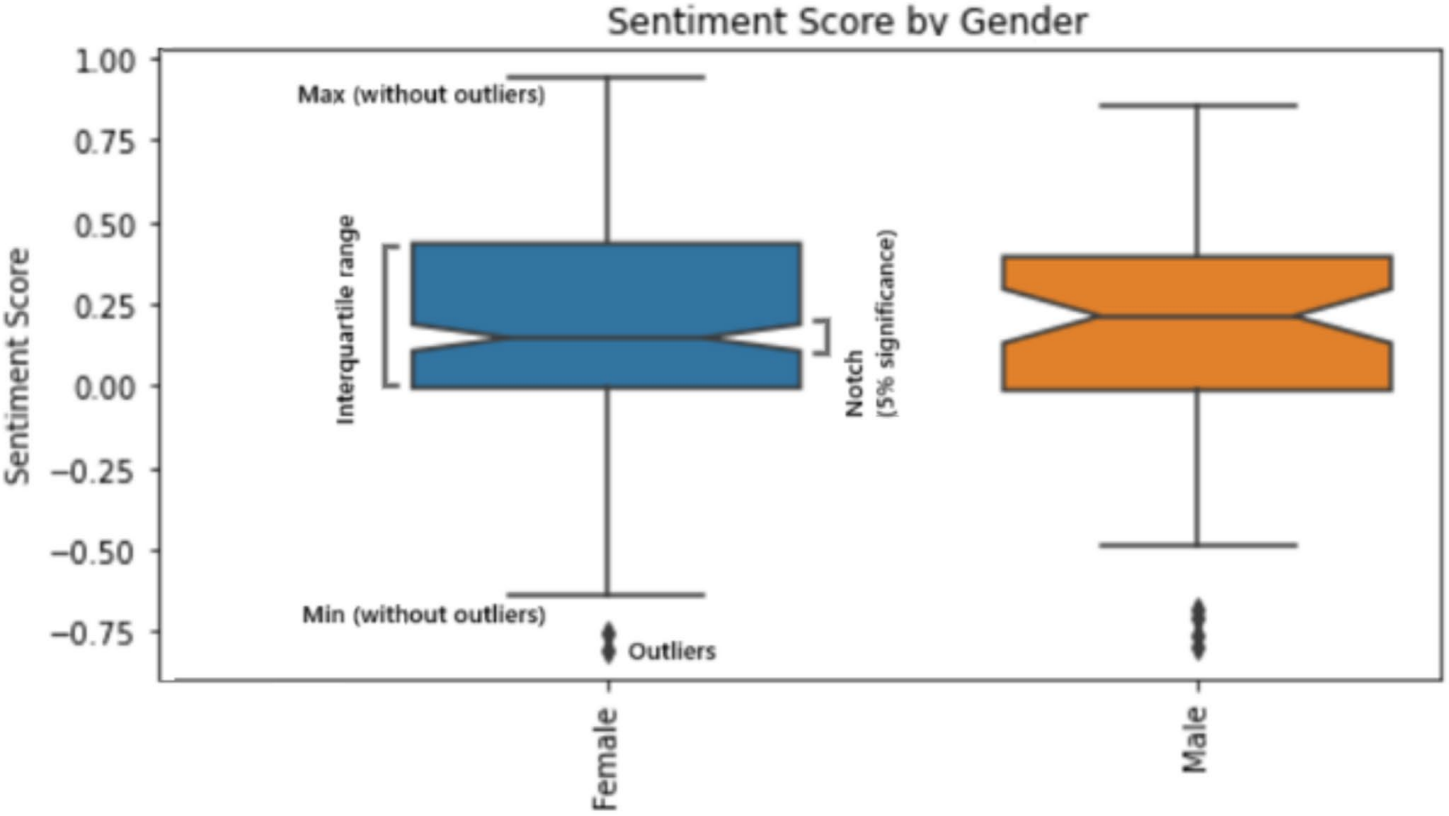
Participants sentiment towards AI by gender (*p* < 0.001).

Male respondents (M = 3.67, SD = 1.07) tend to evaluate their AI competence higher compared to female respondents (M = 3.41, SD = 1.08). However, median values are equal for both genders. Analysis of sentiment among different genders allows to conclude that female respondents have more diverse sentiment towards AI compared to male respondents and have higher mean value but lower median (Figure 3).

Analysis of participants AI competence, trust in AI and sentiment towards AI in public and private sector allows to conclude that there is statistically significant difference between private and public sector employees AI competence (Table 5).

**Table 5:** Comparison of participants AI competence, trust in AI and sentiment towards AI rankings by work sector (Mann–Whitney U test).

Item	Working sector	Mean	Median	Std. dev	*N*	Mean rank	Sum of ranks	U	*P*	η^2^
AI competence	Private sector	3.70	4	1.13	154	971	149,458	102,563	0.002	0.006
	Public sector	3.41	4	1.08	1,559	846	1,318,583			
Trust in AI	Private sector	2.40	2	0.71	154	874	134,612	117,409	0.616	<0.001
	Public sector	2.38	2	0.62	1,559	855	1,333,429			
Sentiment towards AI	Private sector	0.18	0	0.16	26	168	4,358	4,007	0.603	0.001
	Public sector	0.21	0	0.18	328	178	58,477			

**Figure 4:**
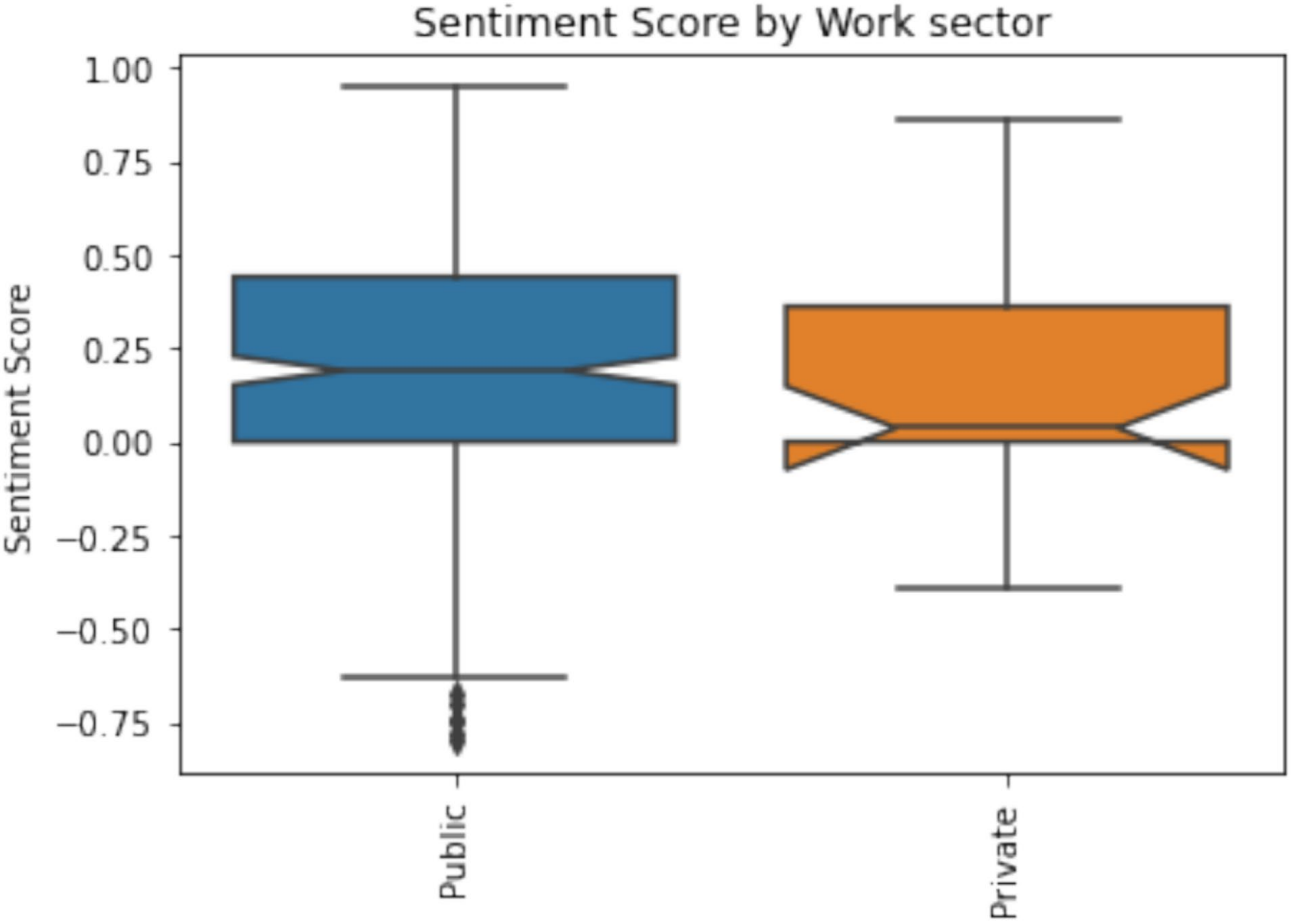
Participants sentiment towards AI by work sector (*p* = 0.001).

Participants from the private sector (M = 3.70, SD = 1.13) tend to assess their AI competence higher compared with participants from the public sector (M = 3.41, SD = 1.08). It indicates that the public sector is lagging behind with innovation implementation. There are no statistically significant differences between Trust in AI and sentiment towards AI among public and private sector workers. However, participants from the public sector have more positive sentiment towards AI compared to participants from the private sector as both mean and median values are higher (Figure 4). Meaning that public sector employees are emotionally as open for AI as employees from the private sector.

Analysis of participants AI competence, trust in AI and sentiment towards AI by education level indicates that there are statistically significant differences for trust in AI by different education groups (Table 6).

**Table 6:** Comparison of participants AI competence, trust in AI and sentiment towards AI self-assessment rankings by education (Kruskal-Wallis H test).

Item	Education	Mean	Median	Std. dev	*N*	Mean Rank	Kruskal-Wallis H	df	P	η^2^
AI competence	Secondary education	3.33	3.5	1.21	58	808	5.302	2	0.071	0.002
	Vocational education	3,23	3	1.10	98	758				
	Higher education	3.48	4	1.08	1,554	863				
Trust in AI	Secondary education	2.22	2	0.70	58	759	6.918	2	0.031	0.003
	Vocational education	2.28	2	0.62	98	772				
	Higher education	2.40	2	0.63	1,554	864				
Sentiment towards AI	Secondary education	0.09	0	0.17	12	142	2.144	2	0.342	<0.001
	Vocational education	0.26	0	0.21	24	193				
	Higher education	0.20	0	0.18	317	177				

**Figure 5:**
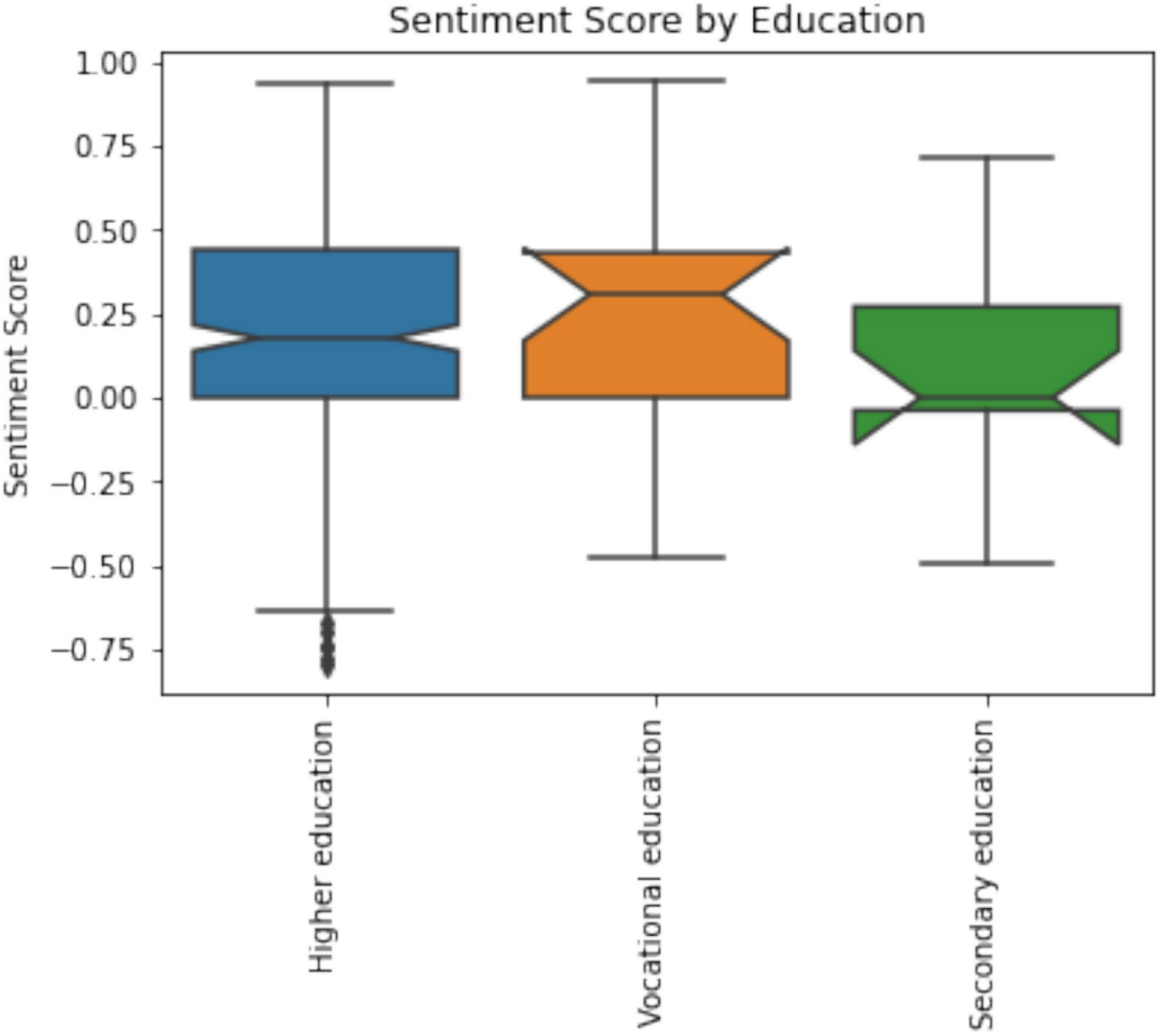
Participants sentiment towards AI by education (*p* < 0.001).

Participants with higher education trust in AI is higher compared to participants with secondary education. However, comparison between secondary education groups indicates that results in trust in AI differs from AI competence as students with vocational education trust information generated by AI more compared to participants with generic secondary education. It might be connected with critical thinking skills nevertheless further research is required. Difference between AI Competence by education level is not statistically significant. However, respondents with academic education have better AI competences. Participants with higher education (M = 3.48, SD = 1.08) have assessed their AI competence higher compared to participants with secondary education and further participants with generic secondary education (M = 3.33, SD = 1.21) have higher AI competence compared to participants with vocational education (M = 3.23, SD = 1.10). However, participants with generic secondary education AI competence are more diverse as standard deviation is highest among education levels. Difference between sentiment towards AI by education levels is not statistically significant. However, participants with secondary education have less positive sentiment compared to other education levels (Figure 5).

Analysis of participants’ AI competence, trust in AI and sentiment towards AI by participants’ age indicates that age influences AI competence and trust in AI (Table 7).

**Table 7:** Comparison of participants AI competence, trust in AI and sentiment towards AI self-assessment rankings by age group (Kruskal-Wallis H test).

Item	Age group	Mean	Median	Std. dev	*N*	Mean Rank	Kruskal-Wallis H	df	*P*	η^2^
AI competence	18–29	4.16	4	1.00	124	1,154	144.232	4	<0.001	0.083
	30–39	3.77	4	1.05	351	990				
	40–49	3.50	4	1.02	469	877				
	50–59	3.23	3	1.07	505	750				
	60–74	3.05	3	1.02	255	675				
Trust in AI	18–29	2.49	3	0.60	124	923	28.173	4	<0.001	0.014
	30–39	2.48	3	0.59	351	925				
	40–49	2.42	2	0.63	469	877				
	50–59	2.30	2	0.63	505	795				
	60–74	2.29	2	0.65	255	787				
Sentiment towards AI	18–29	0.28	0	0.17	16	196	8.466	4	0.076	0.013
	30–39	0.24	0	0.19	62	189				
	40–49	0.23	0	0.17	100	189				
	50–59	0.13	0	0.16	110	155				
	60–74	0.22	0	0.20	65	181				

**Figure 6:**
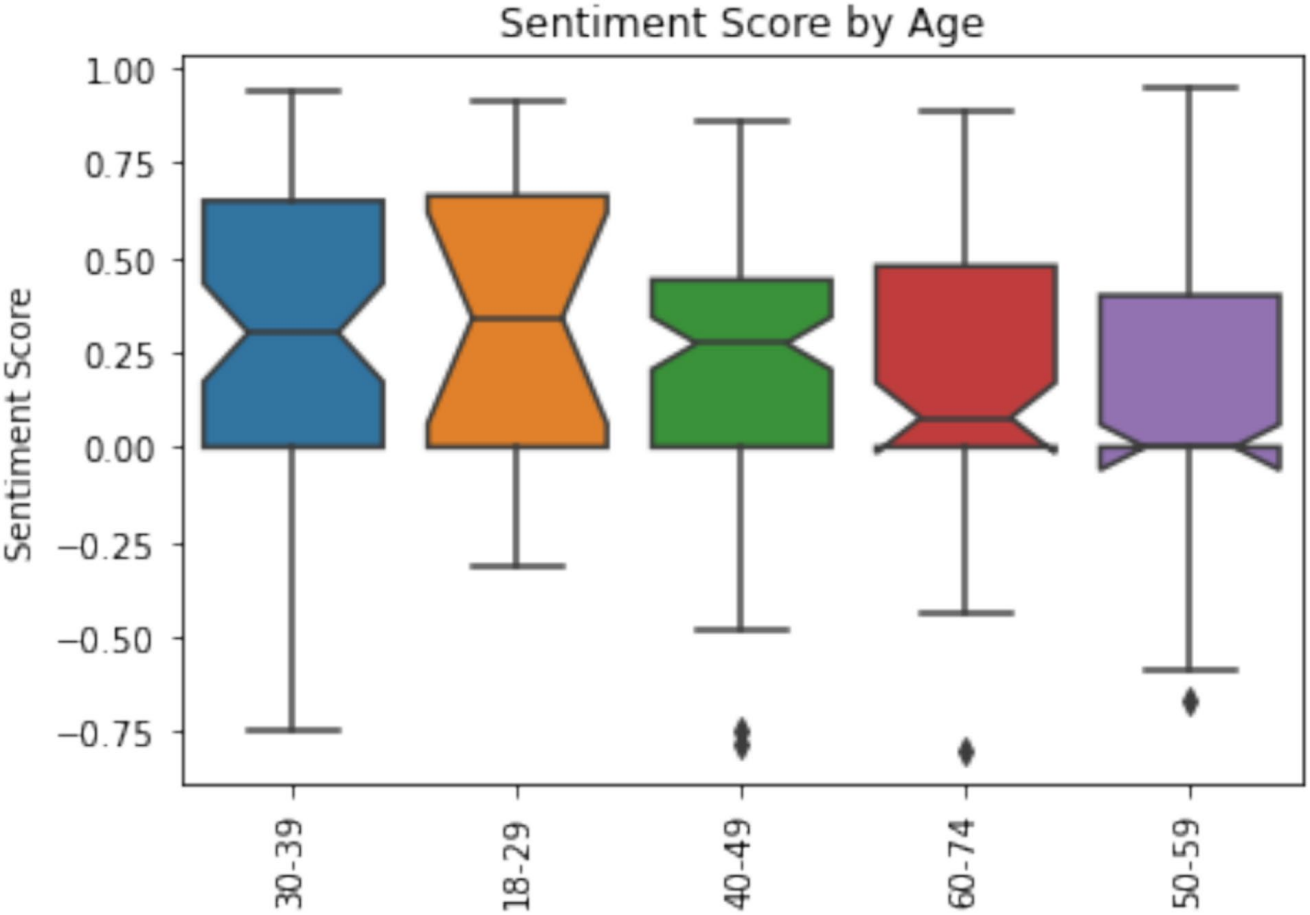
Participants sentiment towards AI by age group (*p* = 0.013).

Younger participants have assessed significantly higher their AI competence. It indicates that older participants are slower to adapt to new innovations. Till age of 49 participants median value is 4 meaning that more than half of respondents deliberately analyze their daily work and select the most appropriate AI tools while for respondents over 50 median value drops to 3 meaning that half of the respondents have only tried simple AI functions and potentially are under higher threat of security risks and disinformation. Similar tendency can be observed with trust in generated information by AI. Younger generation tends to trust AI more compared to older participants. While, at least half of 18–39 year old participants rather trust AI more than half of 40–74 year old participants rather not trust AI. Sentiment analysis does not show clear tendency (Figure 6) and differences between age groups are not statistically significant.

It indicates that older participants feel positive sentiment about AI, but they just lack the competences working with AI and maybe because of that also trust AI less.

### Qualitative research

**Figure 7:**
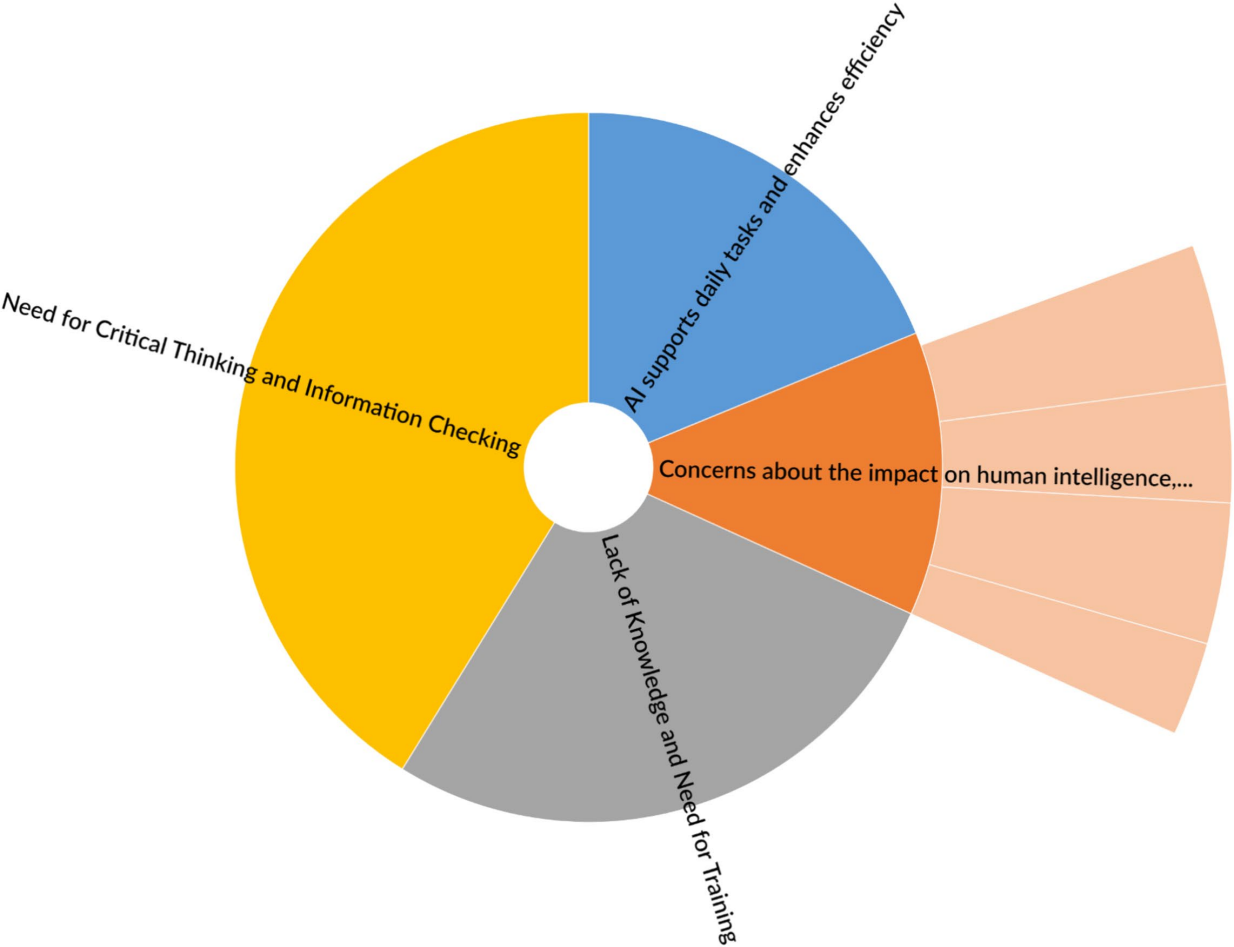
Comparison based on the number of coding references.

At the end of the survey, an open-ended comment section was provided for respondents who wished to share additional thoughts. The responses were coded and analysed using NVivo software, following an inductive approach. Coding is the process of identifying and recording one or more discrete text segments or other data elements that, in some way, represent the same theoretical or descriptive idea (Gibbs, 2002). In NVivo, coding is carried out by linking each segment or item to a node. During the coding process, data segments are typically assigned meaningful labels (Charmaz, 2006). In the early stages of qualitative data analysis, initial or open coding is used to subject the text to intensive examination (Corbin and Holt, 2005). As a result of this process, four codes received the highest number of references (i.e., comments referring to the same theme) (see Figure 7). One of these codes included four child codes, representing different perspectives on the main theme.

The analysis of open-ended survey responses revealed four main thematic codes, each with a different level of prominence based on the percentage of coding references (see Table 8). The most frequently referenced theme was *Need for Critical Thinking and Information Checking* (41.18%), indicating a strong awareness among respondents of the importance of verifying AI-generated information and maintaining critical thinking skills. This was followed by *Lack of Knowledge and Need for Training* (27.06%), highlighting a common perception that users lack adequate understanding of AI and would benefit from targeted education and skill development. The theme *AI Supports Daily Tasks and Enhances Efficiency* accounted for 18.82% of the references, reflecting recognition of AI’s practical benefits in routine and professional contexts. Lastly, *Concerns About the Impact on Human Intelligence, Ethics, and Control* represented 12.94% of the coding references, suggesting that while ethical and cognitive concerns exist, they were raised less frequently compared to themes focused on skills and responsible use. Overall, the distribution of responses underscores both the need for AI literacy and the importance of critical, informed engagement with AI technologies.

**Table 8:** Comparison based on the number of coding references.

Codes	Percentage of coding references
Codes\\AI supports daily tasks and enhances efficiency	18.82%
Codes\\concerns about the impact on human intelligence, ethics, and control	12.94%
Codes\\lack of knowledge and need for training	27.06%
Codes\\need for critical thinking and information checking	41.18%

#### Theme 1: AI supports daily tasks and enhances efficiency

This theme emerged as one of the most prominent in the data, with 18.82% coverage of coding references. Respondents frequently highlighted the practical benefits of AI in their everyday work and personal routines. Many described AI as a “good helper,” a “handy tool,” and a means to “optimize daily work,” particularly for routine or text-based tasks. Some users reported subscribing to advanced tools, such as ChatGPT, and shared examples of using AI to generate images, write texts, or create recipes. Several noted the time-saving potential of AI, emphasizing that it helps complete tasks faster and increases productivity.

However, this generally positive attitude was often accompanied by a cautious awareness of limitations. Respondents acknowledged that AI-generated content is not always reliable, emphasizing the importance of verifying information and applying critical thinking. Comments like *“I always check it before passing it on”* and *“AI does not have all the necessary human qualities, such as intuition and flexibility”* suggest that while AI is viewed as a valuable assistant, it is not perceived as a substitute for human judgment. Some also expressed the need for further education to use AI effectively, especially to avoid misuse or being misled by malicious content.

Overall, the theme reflects a pragmatic and moderately optimistic stance: AI is welcomed as a supportive tool for improving efficiency, provided that users remain critically engaged and informed about its risks and limitations.

#### Theme 2: Concerns about the impact on human intelligence, ethics, and control

Although this theme had the smallest share of coding references (12.94%) among the four identified, it nonetheless reflects a clear thread of apprehension regarding the broader societal and cognitive consequences of artificial intelligence. The code *Concerns about the impact on human intelligence, ethics, and control* included four child codes, each representing a distinct yet interconnected dimension of concern: *AI makes us dull*, *I do not trust AI*, *AI is a threat*, and *AI still needs improvements*.

Respondents frequently expressed fears that excessive reliance on AI could lead to intellectual stagnation and a weakening of human cognitive abilities. Comments such as *“I try to use AI as little as possible so I do not lose my intelligence”* and *“Soon AI will figure everything out for us and our own brains will become dull”* point to a perceived erosion of independent thinking. The theme also reflected a notable lack of trust in AI systems, with participants emphasizing that AI cannot be fully relied upon and should never replace human control: *“AI tools cannot be trusted 100%, as human input will always be needed”*.

Some responses conveyed a more existential concern, positioning AI as a threat not only to knowledge but to societal structures and ethical norms. For example, respondents mentioned AI’s potential to distort reality, dehumanize communication, and enable harmful content creation. Others noted that while AI shows promise, it still requires significant improvement before it can be safely and reliably integrated into critical tasks.

Overall, this theme reflects a deep ambivalence: while recognizing AI’s presence in modern life, many respondents stressed the importance of critical oversight, ethical safeguards, and continuous improvement. The call for education, regulation, and responsible use is clear, indicating that trust in AI is far from automatic — it must be earned through transparency, reliability, and human-centered design.

#### Theme 3: Lack of knowledge and need for training

This theme, accounting for 27.06% of the coding references, reflects a strong and consistent perception among respondents that they lack sufficient knowledge about artificial intelligence and are in need of practical, high-quality training. Across coded responses, participants expressed a desire to better understand what AI is, how it works, and how it can be meaningfully applied in both personal and professional contexts. Many explicitly stated that they “do not know much about AI” or that it remains a “new area” to explore, indicating a widespread gap in foundational knowledge.

A recurring sentiment was the need for accessible and effective training opportunities. Respondents noted that previous training experiences were often superficial or ineffective, and emphasized the importance of hands-on, practical learning formats. Suggestions included in-person group sessions, training with real-world tasks, and sector-specific instruction. Several participants pointed out that publicly funded or workplace-supported training would be particularly beneficial, especially in public administration and education sectors.

Many also linked AI competence to employability and productivity. Comments such as *“Without learning AI, you may no longer be interesting to your employer”* and *“AI must be learned to avoid falling behind”* highlight the perceived urgency to build AI-related skills. Additionally, time constraints were mentioned as a significant barrier, with some expressing frustration that their workloads prevent them from pursuing learning opportunities, despite a strong interest in doing so.

Overall, this theme reveals a widespread readiness and willingness to learn, coupled with a recognition that current knowledge is limited. Respondents called for better support structures—both institutional and societal—to promote AI literacy, empower users, and ensure that the benefits of AI can be harnessed without deepening knowledge gaps or inequalities.

#### Theme 4: Need for critical thinking and information checking

This theme had the highest coverage (41.18%) among all identified codes, underscoring the respondents’ strong awareness of the limitations and risks of artificial intelligence, particularly when it comes to the accuracy, reliability, and trustworthiness of AI-generated content. Across comments, a clear pattern emerged: while many participants acknowledged the usefulness of AI tools, they consistently emphasized the need for human oversight, critical thinking, and independent verification of outputs.

Respondents often described their routine practice of checking and cross-referencing AI-generated information, especially when dealing with sensitive or domain-specific content such as legal, academic, or technical material. Statements like *“I always check,” “Trust, but verify,”* and *“I use tools that provide sources and double-check them”* highlight the practical strategies users adopt to ensure quality and truthfulness. Others noted that AI often lacks context, common sense, and factual accuracy, which reinforces the necessity of human judgment in interpreting and refining outputs.

In addition to concerns about factual correctness, many participants voiced ethical and social worries, particularly about the misuse of AI in spreading disinformation or degrading the quality of communication. For example, one respondent noted, *“Photo, video and audio generators are becoming more natural every day… and will be used for real crimes.”* Another commented, *“Students are trying to solve even the most basic thinking tasks with AI. It’s very bad.”* These insights suggest not only a technical concern but also a cultural and educational one, where critical thinking becomes a key defense against manipulation, misinformation, and intellectual laziness.

The theme also included expressions of uncertainty and skepticism, with several respondents reporting mixed or low trust in AI outputs: *“Confidence 50% – so verifiable,”* and *“I do not trust the information more than 75%.”* Others expressed frustration with the effort required to verify AI-generated content, which sometimes outweighs the benefits of using the tool.

Overall, this theme reflects a mature and discerning approach to AI, where users are neither blindly optimistic nor dismissive. Instead, they show a strong demand for media literacy, critical engagement, and responsible usage, highlighting that successful integration of AI into daily life must include education, transparency, and ongoing human involvement.

## Discussion

This study examined the interplay between AI competence, trust in AI-generated content, and sentiment toward AI among Latvian public and private sector employees. Participants generally rated their own AI competence and trust in AI as relatively high, with most also expressing a positive or neutral sentiment toward AI. These findings are notable given that the widespread use of AI tools has only recently surged. As AI continues to develop rapidly, with new functionalities and risks emerging (Almaiah, 2025), the ability of individuals and institutions to keep pace through continuous competence-building becomes not just a learning goal but a matter of public and digital security.

AI competence enables users to recognize AI-generated content and assess its implications. It is closely linked to trust in AI, which involves evaluating whether AI outputs are credible, contextually appropriate, and aligned with human values (Huang and Ball, 2024). Sentiment, meanwhile, reflects the emotional and attitudinal dimensions of engagement—how comfortable, optimistic, or apprehensive people feel about AI technologies. Taken together, these dimensions illustrate how society is managing the profound opportunities and risks posed by AI.

The study found a positive correlation between AI competence and trust, and between trust and sentiment toward AI. This supports earlier findings that individuals who feel more competent tend to trust AI more, particularly in high-stakes domains (Huang and Ball, 2024; Naiseh et al., 2025). In turn, trust appears to shape whether people feel positively or negatively toward AI. These relationships suggest a reinforcing cycle: higher competence builds trust, which encourages positive emotional responses and acceptance. This dynamic implies that public education and literacy initiatives could be leveraged as tools for both skill development and sentiment management, ultimately improving the safe and ethical uptake of AI in society (Kumar et al., 2025).

Despite relatively high ratings of trust and competence, the majority of participants reported neutral sentiment toward AI. This neutrality may indicate cautious optimism but may also reflect limited emotional investment or uncertainty. Previous survey data show that a significant portion of the population is still more concerned than excited about AI’s growing presence in daily life (Faverio and Tyson, 2023). Such findings highlight the need to promote public engagement with AI that moves beyond technical skills to include real-life examples, narrative case studies, and participatory learning that deepens both understanding and emotional confidence.

The research also revealed that AI competence is higher among private sector employees, while trust and sentiment are comparable across sectors. This difference likely reflects the private sector’s faster pace of digital innovation and exposure to AI tools. In contrast, the public sector’s slower adoption of AI technologies may inhibit competence development, reinforcing findings that training and upskilling efforts remain inconsistent (Gillespie et al., 2025). To ensure digital equity and responsible AI use in governance, targeted investment in AI training for public sector employees is needed—training that goes beyond basic technical skills to address ethical, legal, and societal considerations (Carolus et al., 2023; Almatrafi et al., 2024).

Respondents with higher levels of education showed greater trust in AI, a finding that echoes earlier research linking educational attainment to improved critical thinking, openness to innovation, and digital literacy (Naiseh et al., 2025). Individuals with higher educational level may also be better equipped to evaluate the credibility and limitations of AI-generated information, reducing both undue fear and overreliance. These findings underscore the importance of integrating AI competence into general education curricula, emphasizing not just usage but also ethics, risk awareness, and analytical reasoning (Şahin and Yıldırım, 2024).

Finally, younger participants demonstrated higher AI competence and greater trust in AI—a trend consistent with digital native profiles. These findings point to a potential generational divide in AI preparedness. While younger individuals tend to be more adaptive to technological change (Maslej et al., 2024), older generations may benefit from customized AI literacy interventions that address distinct learning needs and reduce barriers to participation. Ensuring all age groups are included in competence development efforts is vital for building a secure, inclusive digital society.

The findings of this study have clear implications for national and societal security. As AI systems become more deeply embedded in information flows, decision-making, and service delivery, the ability of individuals to competently engage with AI technologies becomes a form of digital resilience. Participants’ generally positive levels of AI competence and trust suggest a promising baseline; however, the widespread neutral sentiment toward AI—combined with uneven competence across sectors and demographic groups—may leave portions of society vulnerable to manipulation, disinformation, or overreliance on unverified AI-generated outputs. As AI tools can be exploited in cybercrime, surveillance, and misinformation campaigns (Almaiah, 2025; Bontridder and Poullet, 2021), ensuring a critically informed and literate public is not only beneficial—it is essential for defending democratic processes, public infrastructure, and digital integrity. Security-oriented AI education that includes threat recognition, ethical awareness, and safe usage practices must become a priority across both public and private sectors.

While the study provides insights into the interplay between AI competence, trust, and sentiment, several areas warrant further exploration. First, longitudinal research is needed to examine how these variables evolve as AI technologies become more widespread and sophisticated. Second, deeper qualitative investigations—such as interviews or focus groups—could complement the survey and sentiment analysis by capturing more nuanced emotional responses and personal experiences with AI. Third, future studies should explore sector-specific needs, particularly within the public sector, where responsible AI implementation intersects with policymaking, transparency, and citizen engagement. Eventually, integrating cross-national comparisons would allow researchers to assess how cultural, institutional, and regulatory contexts influence competence and trust in AI.

## Conclusion

The study explored the relationship between AI competence, trust in AI-generated content, and sentiment toward AI among public and private sector employees in Latvia. The results reveal that while self-assessed AI competence is relatively high, a significant portion of the population either does not use AI at all or uses it only for basic, everyday tasks. This finding highlights a gap between awareness and active, meaningful engagement with AI tools. Furthermore, although the overall sentiment toward AI is mostly positive, a large proportion of respondents remain neutral, suggesting that public attitudes are still evolving.

Statistically significant differences were observed in AI competence across gender, age groups, and work sectors, while trust in AI varied by education and age. In contrast, sentiment was consistent across sociodemographic groups. Importantly, the analysis confirmed that AI competence is positively associated with trust in AI-generated content, and trust, in turn, correlates with sentiment. This dynamic underscores the importance of building both technical and evaluative capacities in society. Enhancing competence and fostering critical trust are essential steps toward promoting safe and responsible AI use.

The thematic analysis further revealed public concerns regarding risk assessment, accuracy, and ethical issues related to AI, as well as uncertainty about AI’s role in everyday life. These reflections suggest that many individuals lack sufficient understanding to assess potential risks, which may weaken society’s ability to critically navigate AI-generated content. Given the growing role of AI in public communication, decision-making, and service provision, these findings are particularly relevant for national and information security.

This research contributes to a broader understanding of how citizens perceive and interact with AI technologies and emphasizes the need for continuous and inclusive competence development. The results offer practical insights for designing public education and workforce training initiatives—especially in the public sector, where digital transformation is slower. Such efforts should address not only technical skills but also ethics, media literacy, and risk evaluation.

Future research should include longitudinal and cross-national studies to track changes in sentiment and competence over time, as well as qualitative approaches to deepen understanding of emotional and cognitive responses to AI. Moreover, targeted strategies should be developed to address the needs of underrepresented groups, particularly older individuals and public sector employees.

Eventually, strengthening AI competence, fostering informed trust, and addressing ethical concerns are key to building a resilient, secure, and forward-looking digital society. As AI technologies continue to evolve, so too must the collective capacity of citizens to engage with them critically and constructively.

### Implications

The findings of this study carry significant implications for education, workforce development, and national security.

1. The positive correlations between AI competence, trust in AI-generated content, and sentiment toward AI support the notion that competence can reinforce trust, which in turn shapes emotional responses and acceptance. This dynamic implies that public education and literacy initiatives can be leveraged as tools not only for skill development but also for shaping sentiment, ultimately improving the safe and ethical uptake of AI.
2. Higher AI competence among private sector employees highlights the need for targeted investment in AI training for public sector employees, going beyond basic technical skills to address ethical, legal, and societal considerations. Demographic patterns—such as greater trust among more educated individuals and higher competence among younger generations—suggest potential divides that must be addressed through inclusive and tailored AI literacy interventions.
3. While trust and competence were generally high, the prevalence of neutral sentiment toward AI suggests cautious optimism or uncertainty. This underscores the need for engagement strategies that go beyond technical instruction to include real-life applications, narrative case studies, and participatory learning that can deepen both understanding and emotional confidence.
4. AI competence is not merely an educational goal; it is a component of digital resilience. Uneven competence across sectors and demographic groups may leave parts of society vulnerable to manipulation, disinformation, or overreliance on unverified outputs. Security-oriented AI education—covering threat recognition, ethical awareness, and safe usage practices—should be prioritized to protect democratic processes, public infrastructure, and digital integrity.

### Limitations

This study also has several limitations that should be acknowledged. First, the self-assessed nature of AI competence may not fully reflect actual skill levels, as individuals tend to over- or underestimate their abilities. Additionally, the single-item measures was used which have lower reliability. Second, while sentiment analysis of open-ended responses offers richer insights than closed-ended items, textual sentiment scoring may miss subtle emotional tones or culturally specific expressions. Third, the sample may not be fully representative of the broader Latvian population, particularly with regard to less digitally engaged or older individuals. Finally, the study focused on general perceptions of AI rather than specific applications (e.g., facial recognition, generative AI, predictive policing), which may elicit different attitudes and security concerns. Addressing these limitations in future work will strengthen the empirical foundation for designing targeted AI education, policy, and trust-building interventions.

## Data Availability

The original contributions presented in the study are included in the article/Supplementary material, further inquiries can be directed to the corresponding author.
