# Supplementary material for: AI competence and sentiment: a mixed-methods study of attitudes and open-ended reflections
Source: Front Artif Intell. 2025 Sep 24;8:1658791. doi: 10.3389/frai.2025.1658791 (PMC12504219; doi:10.3389/frai.2025.1658791)
Supplement: Supplementary file 1 [file Data_Sheet_1.docx]

**Appendix A** AI Competence Questionnaire

| **Question** | **Possible values** |
| --- | --- |
| Please indicate your age | 1- (18-29), 2- (30-39), 3-(40-49), 4-(50-59),5- (60-74), 6-(60-74), 7 - (75+), 8-(Don't want to specify or other) |
| Please indicate your gender | 1- Male, 2- Female, 3-Other |
| Please indicate your place of residence | 1-Capital (Rīga), 2-Cities (Ventspils, Liepāja, Jūrmala, Jelgava, Rēzekne, Daugavpils, Valmiera, Ogre, Jēkabpils), 3-Other towns, 4-Countryside |
| Please indicate your level of education | 1-Basic education, 2-Secondary education, 3-Vocational education, 4-Higher education |
| Please indicate your type of employment | 1- Production; 2- Transport and logistics; 3- Trade; 4- Agriculture, forestry, fishing; 5- Tourism and hospitality; 6- Information technology (IT); 7- Construction; 8- Finance and insurance; 9- Arts, culture and entertainment; 10- Public services; 11- Healthcare and social services; 12- Education; 13- Another |
| Please indicate your employment type | 1- Private; 2-Public |
| Choose the statement that best describes your competence to use MI (artificial Intelligence) | 0 – I haven’t even heard of the relevant AI tools, 1 – I have heard of the tools but haven’t used them, 2 – I know how to use the tools, but have not used them in practice, 3 – I have used the tools for simple tasks, 4 – I analyze my work to deliberately select appropriate AI tools for tasks, 5 – I can evaluate and combine different AI tools for my needs, 6 – I improve or customize AI tools, or integrate them into other systems I use |
| 1. Using AI in question/answer format to obtain factual information | 0 – I haven’t even heard of the relevant AI tools, 1 – I have heard of the tools but haven’t used them, 2 – I know how to use the tools, but have not used them in practice, 3 – I have used the tools for simple tasks, 4 – I analyze my work to deliberately select appropriate AI tools for tasks, 5 – I can evaluate and combine different AI tools for my needs, 6 – I improve or customize AI tools, or integrate them into other systems I use |
| 2. Using AI to generate new text | 0 – I haven’t even heard of the relevant AI tools, 1 – I have heard of the tools but haven’t used them, 2 – I know how to use the tools, but have not used them in practice, 3 – I have used the tools for simple tasks, 4 – I analyze my work to deliberately select appropriate AI tools for tasks, 5 – I can evaluate and combine different AI tools for my needs, 6 – I improve or customize AI tools, or integrate them into other systems I use |
| 3. Using AI for text translation or creating text summaries | 0 – I haven’t even heard of the relevant AI tools, 1 – I have heard of the tools but haven’t used them, 2 – I know how to use the tools, but have not used them in practice, 3 – I have used the tools for simple tasks, 4 – I analyze my work to deliberately select appropriate AI tools for tasks, 5 – I can evaluate and combine different AI tools for my needs, 6 – I improve or customize AI tools, or integrate them into other systems I use |
| 4. Using AI to convert speech or audio documents into text | 0 – I haven’t even heard of the relevant AI tools, 1 – I have heard of the tools but haven’t used them, 2 – I know how to use the tools, but have not used them in practice, 3 – I have used the tools for simple tasks, 4 – I analyze my work to deliberately select appropriate AI tools for tasks, 5 – I can evaluate and combine different AI tools for my needs, 6 – I improve or customize AI tools, or integrate them into other systems I use |
| 5. Using AI to create videos | 0 – I haven’t even heard of the relevant AI tools, 1 – I have heard of the tools but haven’t used them, 2 – I know how to use the tools, but have not used them in practice, 3 – I have used the tools for simple tasks, 4 – I analyze my work to deliberately select appropriate AI tools for tasks, 5 – I can evaluate and combine different AI tools for my needs, 6 – I improve or customize AI tools, or integrate them into other systems I use |
| 6. Using AI to create audio | 0 – I haven’t even heard of the relevant AI tools, 1 – I have heard of the tools but haven’t used them, 2 – I know how to use the tools, but have not used them in practice, 3 – I have used the tools for simple tasks, 4 – I analyze my work to deliberately select appropriate AI tools for tasks, 5 – I can evaluate and combine different AI tools for my needs, 6 – I improve or customize AI tools, or integrate them into other systems I use |
| 7. Using AI to identify objects in images or videos, and to tag or classify them | 0 – I haven’t even heard of the relevant AI tools, 1 – I have heard of the tools but haven’t used them, 2 – I know how to use the tools, but have not used them in practice, 3 – I have used the tools for simple tasks, 4 – I analyze my work to deliberately select appropriate AI tools for tasks, 5 – I can evaluate and combine different AI tools for my needs, 6 – I improve or customize AI tools, or integrate them into other systems I use |
| 8. Using AI for motion or facial recognition | 0 – I haven’t even heard of the relevant AI tools, 1 – I have heard of the tools but haven’t used them, 2 – I know how to use the tools, but have not used them in practice, 3 – I have used the tools for simple tasks, 4 – I analyze my work to deliberately select appropriate AI tools for tasks, 5 – I can evaluate and combine different AI tools for my needs, 6 – I improve or customize AI tools, or integrate them into other systems I use |
| 9. Using AI to make predictions, such as sales forecasts, consumer behavior, or to analyze other quantitative data and make data-driven decisions | 0 – I haven’t even heard of the relevant AI tools, 1 – I have heard of the tools but haven’t used them, 2 – I know how to use the tools, but have not used them in practice, 3 – I have used the tools for simple tasks, 4 – I analyze my work to deliberately select appropriate AI tools for tasks, 5 – I can evaluate and combine different AI tools for my needs, 6 – I improve or customize AI tools, or integrate them into other systems I use |
| 10. Using AI to automate routine tasks, such as organizing and formatting data according to a template | 0 – I haven’t even heard of the relevant AI tools, 1 – I have heard of the tools but haven’t used them, 2 – I know how to use the tools, but have not used them in practice, 3 – I have used the tools for simple tasks, 4 – I analyze my work to deliberately select appropriate AI tools for tasks, 5 – I can evaluate and combine different AI tools for my needs, 6 – I improve or customize AI tools, or integrate them into other systems I use |
| Are there AI training available in your workplace? | 1-No, 2-Rather no, 3-Rather yes, 4-Yes |
| Do you trust the information generated by AI? | 1-No, 2-Rather no, 3-Rather yes, 4-Yes |
| Comments | text |
| Sentiment alculated for comments in "Comment" section | calculated sentiment values between -1 and 1 |
